# An integrated microfluidic device for rapid and high-sensitivity analysis of circulating tumor cells

**DOI:** 10.1038/srep42612

**Published:** 2017-02-15

**Authors:** Jianing Jiang, Hui Zhao, Weiliang Shu, Jing Tian, Yuqing Huang, Yongxin Song, Ruoyu Wang, Encheng Li, Dennis Slamon, Dongmei Hou, Xiaohui Du, Lichuan Zhang, Yan Chen, Qi Wang

**Affiliations:** 1 Department of Respiratory Medicine, The Second Hospital Affiliated to Dalian Medical University, Dalian 116027, China; 2 Shenzhen Institutes of Advanced Technology, Chinese Academy of Sciences, Shenzhen 518055, China; 3 LABVIV Technology(Shenzhen) Co., Ltd, Shenzhen 518055, China; 4 College of Marine Engineering, Dalian Maritime University, Dalian 116026, China; 5 Department of Respiratory Medicine, Affiliated Zhongshan Hospital of Dalian University, Dalian 116001, China; 6 Department of Medicine, Division of Hematology Oncology, Medical School of University of California at Los Angeles, Los Angeles 90095, CA, USA

## Abstract

Recently there has been a more focus on the development of an efficient technique for detection of circulating tumor cells (CTCs), due to their significance in prognosis and therapy of metastatic cancer. However, it remains a challenge because of the low count of CTCs in the blood. Herein, a rapid and high-sensitivity approach for CTCs detection using an integrated microfluidic system, consisting of a deterministic lateral displacement (DLD) isolating structure, an automatic purifying device with CD45-labeled immunomagnetic beads and a capturing platform coated with rat-tail collagen was reported. We observed high capture rate of 90%, purity of about 50% and viability of more than 90% at the high throughput of 1 mL/min by capturing green fluorescent protein (GFP)-positive cells from blood. Further capturing of CTCs from metastatic cancers patients revealed a positive capture rate of 83.3%. Furthermore, our device was compared with CellSearch system via parallel analysis of 30 cancer patients, to find no significant difference between the capture efficiency of both methods. However, our device displayed advantage in terms of time, sample volume and cost for analysis. Thus, our integrated device with sterile environment and convenient use will be a promising platform for CTCs detection with potential clinical application.

Cancer has become a major public health problem, due to its association with most number of patient deaths worldwide^1^. Primarily, it involved tumor cell metastasis^2^,^3^, wherein tumor cells escape the primary lesions, and penetrate into lymphatic or blood vessel system, through which they migrate comfortably to distant places and form metastatic colonies^4^. Circulating tumor cells (CTCs) are defined as tumor cells typically present in the body circulating system. They have generally been considered as “liquid biopsies”, which can be used as a minimally invasive method for diagnosis and assessing cancer status, estimating prognosis, evaluating efficacy and instructing personal therapy^5^,^6^,^7^. However, there has been a tremendous challenge in detecting and capturing these CTCs due to their extreme rarity, with the presence of only 1 to 100 CTCs in 10^9^ blood cells^8^.

Apparently, in the past decade, numerous methods have been developed to isolate the CTCs population based on their properties, particularly their physical or biological properties that differentiate them from blood cells. These mainly include size filtration, density gradient and di-electrophoresis^9^,^10^,^11^,^12^,^13^, which are label-free, convenient and low cost, but have some limitations. For instance, in size filtration method, the clogging due to other blood cell types, makes CTCs capture difficult^14^. Recently the immune-based CTCs separation method involving the heterogeneous expression of surface markers, such as epithelial cell adhesion molecule (EpCAM) has been shown to be useful^15^. The CellSearch system is the only commercial detection system approved by the US Food and Drug Administration (FDA), which uses ferro-fluids conjugated with anti-EpCAM antibody, to magnetically enrich CTCs^16^, and has already been used in the clinic to monitor the response of patients with breast, prostate, and colorectal cancers to specific treatments. Nevertheless, a multi-institutional study involving 177 patients with measurable metastatic breast cancer, reported that CTCs were detected in only 61% of the patients by this system^17^. This has mainly been attributed to the heterogeneous expression of EpCAM^18^,^19^,^20^,^21^,^22^,^23^,^24^. Despite the presence of many potential approaches to isolate and purify CTCs, there several issues must be addressed to realize the full potential of CTCs as a diagnostic and research tool.

Thus, to overcome the issues, microfluidic technology, which literally represents lab on a chip, and has the advantages of high throughput, integration, adjustment and management, with low cost and small volume, may solve the problems. Until now, various CTCs detection methods using microfluidic system have been analyzed, with different cell separation mechanisms, such as size or deformability based isolation, dielectrophoresis, affinity chromatography and magnetic forces^25^,^26^,^27^,^28^. These physical properties based separation systems offer the advantages of label-free sorting, high throughput, and low cost^29^,^30^. However, these methods often results in poor capture rate and poor purity by ignoring the interference of WBCs, and CTCs are also susceptible to damage from large mechanical stresses^27^. Moreover, the immune-based methods to detect CTCs also display few limitations. For instance, it costs too much time for CTCs to react with the antibodies coated on the chip due to the interference and hindrance by other blood components. Subsequently, these factors result in low capture efficiency. Thus, if blood samples could somehow be processed early, then affinity based capturing of CTCs can yield better results.

Hence, our group has developed an integrated microfluidic system that has the potential to overcome the obstacles of any individual method. In the past, we developed an integrated microfluidic system, that included a DLD and a fishbone structure^10^. However, to avoid clogging, blood had to be diluted before adding to the microchannels of the DLD structure. And some WBCs might get stuck in the corners of the fishbone structure. In this study, we have tried to improve the DLD structure, where blood samples needn’t to be diluted to have more convenient and rapid detection. In addition, we have also tried to develop an automatic magnetic purifying device for negative isolation, which decrease the false positive rate to a greater extent, and enhance CTCs purity. In fact with our developed method, CTCs will be captured by the sequential elimination of blood cells in the novel method, where nearly all RBCs and platelets and more than 90% of the WBCs will be first depleted by DLD isolating chip. The remaining small percentage of WBCs will be purified and removed by the automatic immunomagnetic purifying device. Finally, CTCs will be captured on a platform coated with rat-tail collagen, where CTCs are distinguished based on the immune affinity^31^. The integrated system has demonstrated its usefulness not only in artificially spiked blood samples, but also in clinical samples obtained from various advance cancer patients. We observed a capture rate of around 90%, capture purity of about 50% at the high throughput of 1 mL/min, and the viability of CTCs detected through this method reached more than 90%. Furthermore capturing of CTCs using the novel device specifically from the peripheral blood of patients with various advanced metastatic cancers revealed a positive capture rate of 83.3%. In addition, the efficiency of the novel device was compared with CellSearch system, and we observed equal and excellent capture efficiency just as it. What’s more, the results of CTCs detection were in accordance with the clinical condition. Nevertheless, the novel device displayed the advantages in terms of less time, volume and cost required for the analysis. Finally, it is an integrated system which is automatic, sterile and has the convenience of manipulation for universal adaptation, based on the requirement.

## Results

### Design and functioning of the combined microfluidic chip

**Figure 1:**
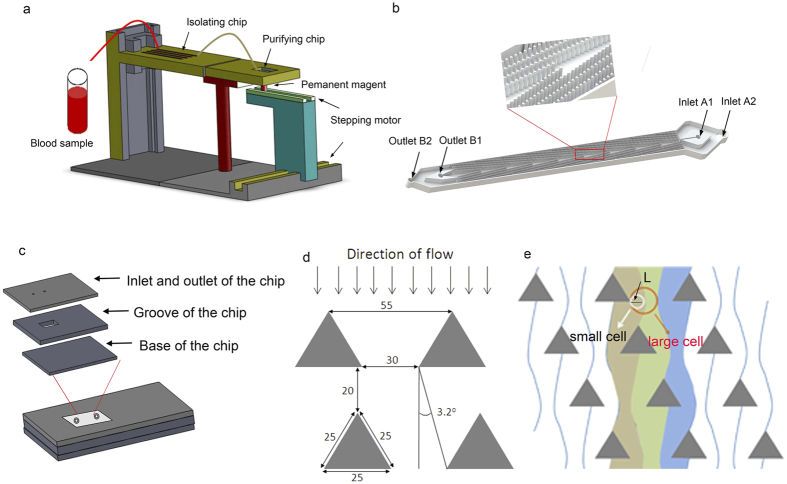
Schematic illustration of the integrated microfluidic device. (**a**) Schematic illustration of the integrated device included isolating chip and the purifying device. The purifying device consisted of purifying chip, the permanent magnet and the stepping motor. Blood sample was pumped into the isolating chip for a preliminary isolation and then passed on to the purifying chip, where CD45 dynabeads were added, and WBCs were eventually removed by the magnet. (**b**) Schematic illustration of the DLD chip. The microfluidic deterministic lateral displacement (DLD) chamber consisted of mirrored triangular micropost array. The large cells (cancer cells and part of leukocytes) from the blood would concentrate in the center of chamber, while small cells (erythrocytes and most of leukocytes) will follow the streamline direction. (**c**) Schematic illustration of the cancer cell purifying chip which consisted of three layers, including a layer for inlet and outlet, a layer for the groove for purified cells and a layer for the base. (**d**,**e**) Schematic illustration of cell separation in DLD micropost array. The fluid (blood sample) flowing through the DLD structures was partitioned into different streams, in which large cells (including cancer cells and some of the leukocytes) flowed in a bumping mode and thus concentrated in the center of the chamber, while small cells (erythrocytes, platelets and most of the leukocytes) followed the direction of the flow.

The newly developed DLD chip was shown in (Fig. 1b). We introduced one chamber with dimensions of 68 mm long, 3.5 mm wide and 50 μm high, two inlets, and two outlets, which was instead of the 8 parallel chambers with dimensions of 42 mm long, 3.5 mm wide and 30 μm high, only one inlet and outlet in the previous design. Moreover, the size of the microarray was also optimized. The new microarray consisted of microposts with 25 μm radius, 30 μm horizontal gaps and 20 μm vertical gaps, with a tilted angle of 3.2 degree towards the fluid flow direction. The cell separation mechanism in DLD array was based on the interaction of cells with microposts under low Reynolds number conditions. As shown in (Fig. 1d,e), microposts can divide the flow of fluid into several narrow streams, and when cells pass through the gaps in microposts, smaller cells with radius less than the width of first stream L, are able to continue following the stream. On the contrary, large cells with radius larger than the width of first stream L, are forced to follow the second stream with a deterministic path. Based on this analysis, 2L can be regarded as the critical cells size. According to the theory proposed by *Loutherback et al.*^32^, the critical separation size of our DLD structure was 6.75 μm. Compared with the previous design, the optimization of the new structure led us to isolate blood samples without diluting with a high throughput of about 1 mL/min, and 3 mL blood would be detected within 3 min. After the first isolation, CTCs with the residual WBCs were collected and subjected to further purification.

Next, a negative isolation based automatic magnetic purifying device was developed instead of the fishbone chip labeled with anti-EpCAM antibody. The size of chip was based on the volume of the samples, and as 200 μL fluid was collected from DLD from 1 mL of blood volume, we applied 600 mm^3^ for patients blood samples purifying. The chip chamber was fabricated with a 20 (Length) × 10 (Width) × 3 (Height) mm^3^ size, and a corresponding computer code was written to move the magnet within the borders of the chamber. The movement of the magnet was in a rectangular shape, as it moved from one length to another and one width to another, with a round trip.

**Figure 2:**
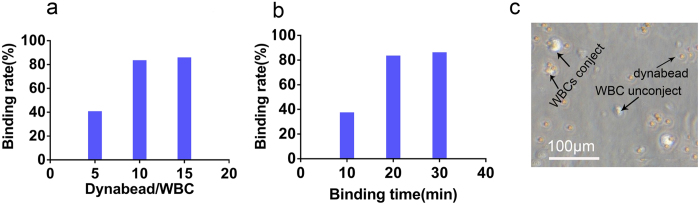
The binding rate of the purifying device under different ratios of dynabead/WBC and different binding time. (**a**) When the binding time was 20 minutes, at different ratios, the different binding rate was shown. (**b**) When the ratio was 10:1, at different time points, the different binding rate was shown. (**c**) The picture showed when CD45 dynabeads were mixed with WBCs for 20 minute at 10:1 ratio, magnification, X200. The smaller spheres were CD45 dynabeads with the diameter of 4.5 μm, while the bigger size structures were WBCs isolated from the peripheral blood of healthy donors.

In addition, the best parameters were also standardized, including the binding rate (the number of WBCs binding to CD45 dynabeads/all the WBCs) and the binding time. The human WBCs were isolated from the healthy volunteers by using RBC Lysis Buffer (1:10, eBioscience) and CD45 immunomagnetic dynabeads (Invitrogen, USA). As the recommended ratio and time were 5:1 (Dynabeads/WBCs) at 30 min, different ratios (5:1, 10:1, 15:1) and different time (10 min, 20 min, 30 min) points were tried. The binding time of 20 min displayed the binding rate of 40.09% (5:1), 82.95% (10:1) and 85.25% (15:1), (Fig. 2a). However at a ratio of 10:1, the binding rate was 36.9% (10 min), 82.95% (20 min) and 85.6% (30 min), (Fig. 2b) Finally, the 10:1 ratio and 20 min time were identified as best parameters, and under this condition, WBCs were binding to the dynabeads sufficiently (Fig. 2c). Further, to verify the advantage of the novel developed device, the similar experiments was performed with the traditional method using DH-II Rotary mixing apparatus (Xinzhi, Ningbo, China) to remove WBCs, and observed that removal rate with the traditional method was about 10% lower than the automatic device.

### Analysis of the capture efficiency of lung cancer cell line by the novel device

**Figure 3:**
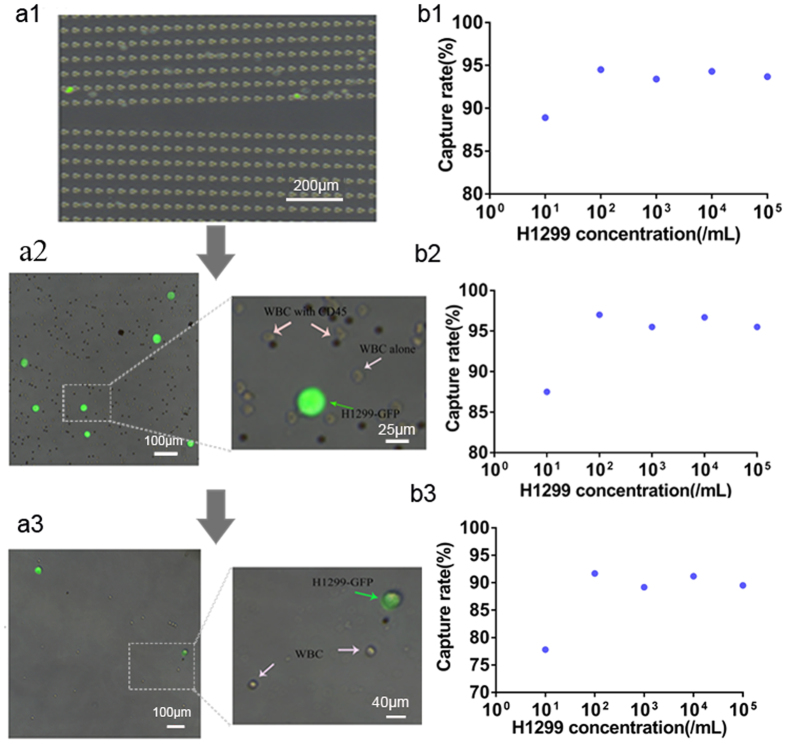
Capture rate of H1299-GFP cells in the novel device (**a1**–**a3**) showed merged bright and fluorescent field images of cancer cells (H1299-GFP) and blood cells in the isolating system (**a1**) (magnification, X100), purifying system (**a2**) and capture system (**a3**) (magnification, X200). (**b1**–**b3**) showed capturing rate of different concentrations of H1299 cancer cells at DLD (**b1**); at CD45 immunomagnetic device (**b2**); and in the integrated device (**b3**). The original concentration of H1299 cancer cells in (**a1**–**a3**) was 10^3^/mL.

To mimic the capturing of actual CTCs from the blood samples, we generated a model where peripheral blood of healthy volunteer was spiked with H1299-GFP cell line at concentrations of 10^1^ to 10^5^ cells/mL. The spiked blood samples were introduced smoothly into the integrated system, (Fig. 3a1–a3). The GFP expression in H1299 cells led us to easily track these cells from rest of the blood cells. The capture rate and capture purity were two important parameters tested to evaluate the system. We separately calculated the capture rate of DLD isolating chip, the automatic purifying device and the integrated system, respectively. The DLD isolating chip showed the H1299-GFP cells capture rate of 88.9%, 94.5%, 93.4%, 94.3% and 93.7% among the blood when spiked with a concentration of 10^1^/mL to 10^5^/mL, respectively (Fig. 3b1). Similarly, the capture rate of automatic magnetic device was 87.5%, 97.0%, 95.5%, 96.7% and 95.5% respectively for different concentrations (Fig. 3b2). Moreover, the integrated system displayed a capture rate of 77.8%, 91.7%, 89.2%, 91.2% and 89.5%, respectively for similar concentrations of H1299-GFP cells (Fig. 3b3). Overall, the capture rate was around 90%. In terms of capture purity, we observed about 50% at the 10^3^/mL concentration, and it increased in parallel with increase in H1299-GFP cell concentration.

### Analysis of the viability of captured H1299-GFP cancer cells

**Figure 4:**
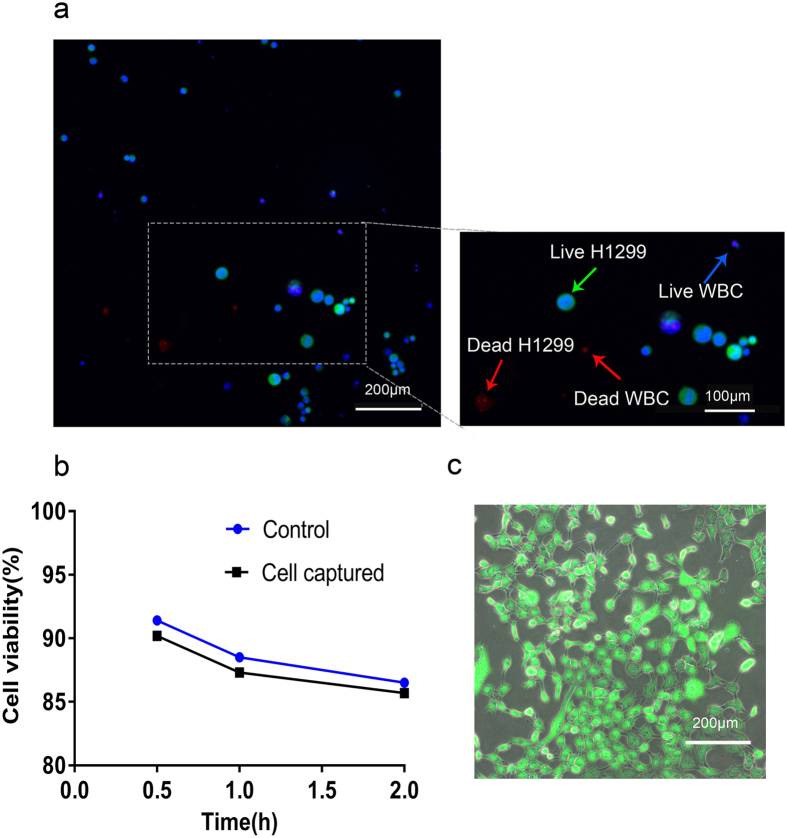
Cell viability of captured H1299-GFP cells. (**a**) The viability of captured cells were evaluated using a fluorescent LIVE(blue)/DEAD(red) assay. Live H1299-GFP cells showed co-localization of green and blue colors. The live WBCs showed only blue color. Scale bars 200 μm and magnification, X100. (**b**) Cell viability of H1299-GFP cells spiked in blood. Blue bars represented control cells, while black bars showed captured and released cells. (**c**) Released H1299-GFP cells showed proliferation and maintained viable after five days of culturing, as recorded by optical microscope at magnification, X100. The original concentrations were 10^3^/mL in (**a**) and 10^4^ in (**c**).

Next, the viability of the captured H1299-GFP cells were measured, using Hochest/PI assay (Fig. 4a). The numbers of the viable H1299 cells in the blood samples were observed and counted under microscope, and later compared with the cells cultured in 24 well plates at room temperature. The results showed that captured H1299 cells had viability of 90.2% after 30 minutes culturing in a plate, but it decreased to 87.3% after 1 hr and 85.7% after 2 hrs of culturing at 37 °C. This rate was similar to the cell viability of the control cells that did not pass through the integrated system. The viability of the control cells was 91.4% after 30 min, while it reduced to 88.5% after 1 hr and 86.5% after 2 hrs (Fig. 4b). Further culturing of H1299 cells captured via the integrated device at 37 °C with 5% CO_2_, led them to adhere to the surface of the plate and proliferate similar to the control cells, and thus suggesting that these cells were not damaged (Fig. 4c). The expression of green fluorescence further indicated that these originally spiked cells into the whole blood, can survive many generations.

### Detection of CTCs from patient blood samples

**Figure 5:**
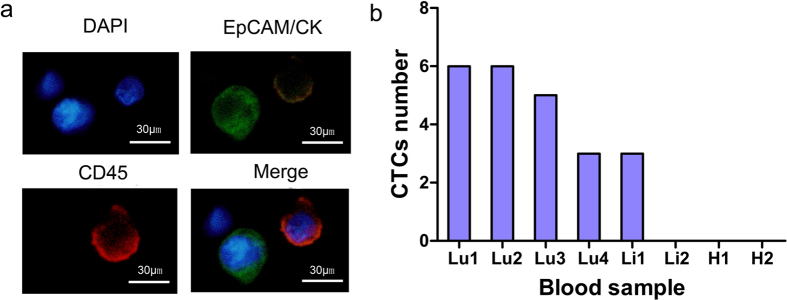
Analysis of captured CTCs from blood samples of patients. (**a**) The representative immunofluorescent staining images of the cells captured from the patient blood samples were shown. The green color represents EpCAM and CK protein expression, blue color showed nuclear staining (DAPI), and red color depicted CD45 expression. Magnification, X640. (**b**) The total number of CTCs with EpCAM^+^ or CK^+^/DAPI^+^/CD45^−^ phenotype was shown, captured in the blood samples from 6 patients (Lu1 to Li2) and two healthy donors (H1 and H2) using the integrated system.

Finally, we tested the developed integrated system for capturing CTCs from the advanced cancer patients’ blood samples. The six cases of patients with advance cancers, including 4 cases with metastatic lung cancer, and 2 cases with metastatic liver cancer, along with 2 healthy volunteers as controls, were involved in the study. The 3 mL blood sample obtained from each subject for CTCs detection was run through the integrated device. The identity of the captured CTCs was confirmed by the positive expression of EpCAM or CK proteins as shown with a green fluorescent signal, and simultaneous negative expression of blood cell leukocyte marker, CD45, a red fluorescent signal (Fig. 5a). Among the 6 patients, we identified CTCs in 5 patients (83.3% positive detection rate), while the remaining patient showed no CTCs. The average numbers of CTC identified were between 3 and 6, with an average of 3.8/mL of peripheral blood. However, we did not observe any CTCs in both healthy controls (Fig. 5b).

### Comparison of the efficiency of the integrated device with CellSearch System

To further testify the efficiency of the novel device, CTCs detection from 30 patients was compared in parallel with CellSearch System. The basic information of each patient was recorded, including gender, age, diagnosis, tumor stage, treatment. All these parameters along with the comparative analysis results of CTCs by both systems have been summarized in (Table 1). Parallel analysis of blood samples from 30 cancer patients revealed no significant difference between the two methods in terms of CTCs capture efficiency (p = 0.1855). It suggested that the integrated system had equally excellent capture efficiency as CellSearch System. Moreover, from the results, it was found that few CTC was detected in patients at I or II stage. However, 16 in 23 patients at III or IV stage were positive in CTCs detection, with the positive rate 70.0%, which corresponded to the clinical condition.

However, the novel device still displayed the advantage in terms of requiring less volume of blood sample, less expense, and even less time for analysis as indicated in (Table 2).

## Discussion

CTCs acts as “seeds” of incurable metastasis and thus represent a promising and effective alternative to invasive tumor biopsies to detect, monitor and combat solid tumors in patients^33^,^34^,^35^,^36^. However, the presence of very few numbers of CTCs in the blood makes their detection and capture hard and thus hinders the realization of them being an important indicator for diagnosis, prognosis and therapeutic targets of metastatic cancer.

In the past decade, an increasing number of microfluidic devices have been developed to assist efficient CTC isolation and detection^25^,^26^,^31^, and are broadly based on physical and/or immune-based separation^37^. Among physical properties, size-based isolation is the most widely used, which employs size-based membrane filters and size/deformation-based hydrodynamic methods. As alluded earlier, the disadvantages of these methods cannot be ignored. For instance, a study by Li *et al*., used surface-micromachined PDMS microfiltration membrane (PMM) to isolate WBCs from lysed blood. It resulted in the filtration throughput of 1 mL/h with the recovery rate 27.4 ± 4.9%^38^. Thus it consumed much time and resulted in low capture rate. Another study by Loutherback *et al*. developed a deterministic method to separate cancer cells from blood at 10 mL/min rate with the capture rate of 85%^39^. This study although had enough throughput, but the capture rate was not very efficient, and moreover resulted in capturing of similar-sized WBCs along with CTCs. Based on dielectrophoretic (DEP) force, Gupta *et al*. have designed a microfluidic CTC-isolation device that exploited the varying directionality of the DEP force and acted on cancer and blood cells in a suitable electric field with capture efficiency of around 70% and a throughput of 1 mL/h^40^, but it also had some issues like low capture efficiency and throughput. However, on the other side, immune-based separation methods, for example, herringbone-chip instead of microarrays functionalized with antibodies by Li *et al.,* increased the capture rate up to 90%^27^. But, to achieve sufficient contact with the antibodies, the flow rate has to be as slow as 1 mL/h and blood has to be lysed. Thus it would lead to huge volumes and would require too much time.

In this study, we have developed an integrated microfluidic system, which overcame the defects of single platform, had high throughput of 1 mL/min, showed high capture efficiency and excellent viability of about 90%, a fine capture purity of about 50%, in comparison to most of the other developed methods^41^,^42^. Also, this system integrated every single device together as sterile, automatic and more convenient, which not only can be translated to the clinical application, but can also help in further analysis of CTCs by single cell sequencing. In this integrated system, we improved the DLD design to be used rapidly and conveniently in high throughput conditions, without diluting the blood samples. Furthermore, compared to other negative isolation systems^43^, our automatic immumomagnetic purifying device was able to efficiently remove WBCs and exclude their interference in CTCs purification. We showed that 20 min time period was sufficient for WBCs to bind with CD45 dynabeads. The reduced time showed decreased removal rate. When the incubation time was 10 min, the removal rate was further decreased by half. However, in most of the previous approaches, the mixing time was less than 10 min and this can explain the partial removal of WBCs. As we know, the presence of WBCs results in false positive, which subsequently led to misunderstanding of the patient condition, and indicate towards further analysis of CTCs. The novel device has demonstrated the ability to capture pure CTCs. In addition, this device is automatic and small, and it not only substituted for the manual, tedious and heavy method, but also increased the removal rate by 10%.

Next, to assess the efficiency of our integrated device in clinic, we analyzed the peripheral blood of 6 patients with advanced metastatic cancers and 2 healthy volunteers. We reported that 5 out of 6 patients displayed an average of 3.8 CTCs/mL blood with a positive rate 83.3%, which was consistent with the clinical condition. Further analysis of the clinical history of the case, where we fail to capture CTCs, revealed that the patient suffered from liver cancer and had intrahepatic metastases, which could somehow restrict the availability of CTC to peripheral blood.

It is worth mentioning that we also tested the efficiency of the novel device against CellSearch System (Janssen Diagnostics, LLC.) for CTCs detection. The parallel analysis of blood samples from 30 cancer patients revealed that there was no significant difference in the capture efficiency by the two methods, with p value of >0.05. Moreover, it was found that few CTC was detected in patients at I or II stage. However, 16 in 23 patients at III or IV stage were positive in CTCs detection, with the positive rate 70.0%, which corresponded to the clinical condition. And, the view that CTCs occurred in the peripheral blood of patients with metastatic cancer was confirmed again. However, in addition to its less expense, less blood sample required, the novel device was a small integrated device which was sterile, automatic, convenient and easier for universal adaptation^44^. All these benefits may make it more suitable for clinical application. As we know, heterogeneous makes the failure of cancer therapy, which has played a tremendous role and been focused on^45^,^46^,^47^. The novel device with a rapid detection, a high capture efficiency and an excellent vitality, which maintain the heterogeneous, can be delivered to further analysis such as single cell sequencing.

However, there are still several difficulties that need to be addressed. First, CTCs captured from patient blood samples needs to be fixed with paraformaldehyde, and then permeabilized with triton before performing immune affinity reaction, and these steps eventually affect the multiple proteins inside the cell, and leads to difficulty in maintaining the *in vitro* culture just like *in vivo* conditions. Second, despite the depletion of majority of the WBCs by DLD structure and automatic magnet device, less than 1% of them are still left and interfere. Therefore, more refinement in the structure of the device needs to be done to totally deplete all WBCs.

In summary, we have developed a rapid and high-sensitivity device for detecting and capturing CTCs, that consisted of DLD isolating structure, automatic magnetic purifying device and rat-tail collagen coated dish, which demonstrated the capture efficiency of around 90% and a capture purity of about 50%, for cancer cell line which displayed a viability of more than 90% after capture at the high throughput of 1 mL/min. These capture efficiency was evidently better than most previously developed devices. In addition, the novel device has the advantage of automation, integration, convenience and low cost. Furthermore, the novel also detected the CTCs from the blood samples of patients with advanced cancer at a positive rate of 83.3%, which seems to be a very significant in terms of clinical diagnosis, prognosis and therapy. It is particularly worth mentioning that our device has obvious advantages over CellSearch System specifically in terms of time and amount of blood required for analysis. In future, we would like to further extend the utility of our device in carrying out single cell sequencing to address the issue of cancer heterogeneity.

## Materials and Methods

### Microfluidic chip fabrication

This entire device consisted of three parts. The DLD isolating structure and the automatic magnetic purifying device were fabricated on the microfluidic chip (Fig. 1a). The schematic structure of DLD chip and automatic purifying device were shown (Fig. 1b,c). The DLD chip had two inlets, two outlets with triangle pillar posts among them. The chip was fabricated using polydimethylsiloxane (PDMS, Sylgard 184, Dow Corning, Midland, MI, USA) which was molded on a master, prepared by spin-coating a 100-μm layer of SU8-2035 negative photo resist (Microchem Corp., Newton, MA, USA) onto a glass wafer and patterned by photolithography^48^. The Sylgard 184 PDMS base and curing agent were mixed thoroughly in a 10:1 mass ratio, degassed under vacuum, and poured onto the master. The polymer was oven-cured at 80 °C for 1 hr, and after cooling, the PDMS layer was gently peeled from the master and trimmed to specific size. Inlet and outlet holes were punched out of the PDMS to form reservoirs for the introduction of liquid. The PDMS was bonded irreversibly to a glass slide (1.2 mm thick) after 60 s oxygen plasma treatment. Next, the microfluidic device was sterilized under 110 kpa at 120 °C for 0.5 hr and UV sterilized for 30 min before use.

Additionally, similar to the structure developed by Grzybowski *et al*.^49^, an automatic purifying device consisting of a chip, an electronic control system, and a permanent magnet was developed. This chip was fabricated using Polymethylmethacrylate, and the microchannels were created using a Laser Engraving Machine (LS100CO_2_, Gravograph, Shanghai). The chip was fabricated with three layers, which were bonded to each other using a Thermocompressor (CARVER, USA) at 120 °C for approximately 10 minutes. The inlet was connected to the outlet of the DLD device. The outlet was used for adding CD45 dynabeads and for collecting CTCs. The reaction chamber was cuboid in shape. The diameter of the inlet and the outlet were both 1.5 mm, and movement of the magnet was regulated using an electrode controlled by a C Language-based computer program. The diameter of the magnet was 5 mm. The magnet attracted the dynabeads to the bottom of the chamber; thus, the CD45 dynabeads used to bind WBCs were removed with the magnet.

### Rat-tail collagen coating

The standard CTCs purification protocol would leave few WBCs among them and thus they remains to be identified. To accomplish this, rat-tail collagen (0.012 mg/mL, Solarbio, China) was used to coat the chamber, because CTCs, based on antigen-antibody immune affinity, adhered better on the coated surface^50^.

### CTCs identification by immunostaining

The captured cells were analyzed by immunofluorescence staining, where they were first fixed with 4% paraformaldehyde for 10 min. The fixed cells were later permeabilized with 0.25% triton for 10 min. The CTCs were identified using FITC mouse anti-human EpCAM IgG2 (1:10, BioLegend), and Alexa Flour 488 rabbit wide spectrum anti-cytokeratin (1:10, abcam) antibodies. The PE mouse anti-human CD45 IgG1 (1:10, BD Biosciences) was used to distinguish WBCs. The nuclei were labeled with DAPI (1:500, sigma). Cells were observed under fluorescent microscope, and the EpCAM^+^ or CK^+^/DAPI^+^/CD45^−^ phenotype confirmed CTCs, while CD45^+^/DAPI^+^/EpCAM^−^/CK^−^ phenotype represented WBCs.

### Cell line culture and preparation

For the human lung adenocarcinoma cell line NCI-H1299, obtained from the American Type culture collection (ATCC), VA was first stably transfected with green fluorescent protein (GFP) protein (H1299-GFP). This modified cell line was then used instead of actual CTCs as a model to evaluate the reliability of the developed device. The cells were cultured according to the protocols from ATCC in DMEM/HIGH GLUCOSE medium supplemented with 10% FBS and 1% Penicillin/Streptomycin at 37 °C with 5% CO_2._ The medium was changed every 1–2 days of culturing. Cells were trypsinized by incubation in 0.05% Trypsin-ethylene diamine tetra acetic acid (EDTA) (Invitrogen, CA) at 37 °C for 2 min to suspended them and subsequently dilute them to the desired concentration.

### CTCs isolation and capture on cell line circulated in the healthy blood

The blood samples from healthy individuals were provided by the Second Hospital of Dalian Medical University, PR China. The experimental protocol was approved by the Ethics Review Committee of the Second Hospital of Dalian Medical University, and this study was conducted in compliance with ethical and safe research practices involving human subjects or blood. Informed consent was obtained from all subjects. Clinical characteristics and information of the healthy volunteers were obtained from their medical records. All these blood specimens were collected in ethylene diamine tetra acetic acid (EDTA) containing tubes and were processed within 6 hours. In addition, these specimens were spiked with HI1299-GFP stable cells at concentrations from 10^1^ to 10^5^ cells/mL. Next, 5 samples were tested on the integrated system design, by pumping them onto the DLD chip with OB1 Pressure Controller (ELVEFLOW, France). This pressure controller had the advantage over the traditional injection pump, in terms of maintaining uniform force to move the fluid more stably, and also simultaneously providing different forces through different passageways.

First, under pressure exerted by a OB1 Pressure Controller (ELVEFLOW, France), the blood specimens were pumped into the Inlet A2, and PBS was injected into the Inlet A1 to balance the pressure. The section (waste) containing the RBCs and most WBCs was collected at Outlet B2, while CTCs and the residual WBCs passed through Outlet B1, which was subsequently connected to the inlet of the automatic purifying device by a flexible plastic tube (TYGON, USA). CD45 Dynabeads were then added into the purifying chip through the outlet. To improve the interaction between anti-CD-45 Dynabeads and WBCs, we developed the purifying device, in which Dynabeads were forced via magnet to move around the chamber to enhance their interaction with WBCs. Further, to ensure that captured WBCs and Dynabeads were removed, the magnet from the purifying device was allowed to rest at the bottom of the chamber, and the supernatant containing CTCs and uncaptured WBCs was sucked out. Finally, cells were captured on the rat-tail coated dish and counted under microscope. The capture rate and capture purity were calculated with the following equation: Capture rate = C1/N %; Capture purity = C1/C2%; where C1 is the number of cancer cells captured from the chip, and N is the number of all the cancer cells pumped at the starting point, C2 is the number of all the cells (cancer cells and white blood cells) captured from the device.

Next, the viability of the captured cells was measured by a standard Live/Dead fluorescent assay (Hochest/ PI staining), whose result was compared with the control cells that were never introduced into the microfluidic device. Both these cell types were cultured at 37 °C with 5% CO_2_ and the medium was changed every 1–2 days, and the growth and proliferation were observed every day.

### CTCs isolation and capture from metastatic cancer patients

Furthermore, the blood specimen (3 mL) of patients with advanced metastatic cancer, who were recruited at The Second Hospital Of Dalian Medical University, PR China, were collected for CTCs detection after their informed consent. The experimental protocol was approved by the Ethics Review Committee of the Second Hospital of Dalian Medical University, and this study was conducted in compliance with ethical and safe research practices involving human subjects or blood. Clinical characteristics and information of the patients were obtained from their medical records. These blood specimens were also collected into ethylene diamine tetraacetic (EDTA)-containing tubes and processed within 6 hours as outlined in the above paragraph. The clinical history of each patient was also considered, which contributed to the analysis of the results of CTCs detection by the novel device.

### Comparison of the novel device with CellSearch System for capture efficiency

To further test the novel device, more blood samples from advanced metastatic cancer patients, were collected. Each patient donated 10.5 mL of blood for detection, wherein 7.5 mL was processed with Cell Search System, while 3 mL volume was analyzed in the novel device. Samples detected by CellSearch were processed within 96 hrs. Capture efficiency by the two methods were compared by the peripheral blood samples from 30 patients with advanced metastatic cancer. The condition of the each patient, including gender, age, diagnosis and therapy was also recorded, to correlate the results of CTCs detection by the two methods.

### Statistical analysis

All statistical analyses were carried out using SPSS 13.0 statistical software package (Chicago, IL, USA). Data were expressed as mean ± standard deviation (SD). Statistical significance of the differences between experimental groups was determined using Student’s *t*-test. *P* < 0.05 was considered statistically significant.

## Additional Information

**How to cite this article**: Jiang, J. *et al*. An integrated microfluidic device for rapid and high-sensitivity analysis of circulating tumor cells. *Sci. Rep.* **7**, 42612; doi: 10.1038/srep42612 (2017).

**Publisher's note:** Springer Nature remains neutral with regard to jurisdictional claims in published maps and institutional affiliations.

## Figures and Tables

**Table 1:** Comparison of the efficiency between CellSearch System and the novel device.

Patient	Gender	Age	Diagnosis	Stage	Treatment	Count(n)	
						A(7.5 mL)	B(3 mL)
1	Female	57	Breast Cancer	IIB	S,E	0	2
2	Female	64	Colon Cancer	IIIC	S,C	0	16
3	Male	62	Thymoma	IVB	S,C	80	15
4	Female	55	Breast Cancer	IIA	S,C	0	0
5	Male	82	Lung Cancer	IVA	S,C,R	0	0
6	Female	45	Breast Cancer	IA	S	0	0
7	Female	65	Breast Cancer	IIA	S,C	0	0
8	Male	55	Lung Cancer	IIIA	S,C	0	0
9	Male	68	Lung Cancer	IIIB	M	3	6
10	Male	58	Bladder Cancer	IIIA	C	0	9
11	Male	78	Lung Cancer	IVA	S,M	0	0
12	Female	76	Breast Cancer	IIB	S,C	0	0
13	Female	46	Breast Cancer	IIB	S,C	0	0
14	Female	60	Lung Cancer	IIIA	S,C,M	0	0
15	Female	76	Breast Cancer	IIB	S,C,M	0	0
16	Male	60	Colon Cancer	IIIB	S,C	30	15
17	Female	57	Lung Cancer	IVA	C	2	9
18	Male	44	Lung Cancer	IVA	C,M	0	4
19	Male	72	Myeloma	IVB	C	0	0
20	Female	67	Colon Cancer	IVA	C	3	2
21	Male	72	Lung Cancer	IVB	C	35	16
22	Female	63	Lung Cancer	IVB	C	10	5
23	Male	72	Lung Cancer	IVA	C	0	3
24	Male	69	Lung Cancer	IIIB	C,R	3	4
25	Male	64	Colon Cancer	IIIA	S,C	0	1
26	Male	65	Lung Cancer	IIIA	C	64	20
27	Female	70	Breast Cancer	IIIA	S,C	0	2
28	Female	68	Lung Cancer	IVA	C	15	3
29	Female	57	Lung Cancer	IVA	E	0	0
30	Female	72	Myeloma	IVB	C	0	0

Group A represents CellSearch system based CTCs analysis in 7.5 mL peripherial blood, while Group B represnts for the same with the novel device in 3 mL. S denotes surgery, C denotes chemotherapy, E denotes Endocrinotherapy, M denotes Molecular targeting treatment, and R denotes radiotherapy.

**Table 2:** Comparison of Cellsearch System and Microfluidic System in CTCs detection.

Device	Samples required (mL)	Detection Time(h)	Expense (yuan)	Further analysis	Device Portable Volume	Universal
A	7.5	4	5000	No	Huge No	Hard
B	3	1	500	Yes	Small Yes	Easy

“A” stands for CellSearch System, “B” stands for the novel device.
